# Hypouricemic and arthritis relapse-reducing effects of compound tufuling oral-liquid in intercritical and chronic gout: A double-blind, placebo-controlled, multicenter randomized trial

**DOI:** 10.1097/MD.0000000000006315

**Published:** 2017-03-24

**Authors:** Zhijun Xie, Huaxiang Wu, Xiaoqing Jing, Xiuyang Li, Yasong Li, Yongmei Han, Xiangfu Gao, Xiaopo Tang, Jing Sun, Yongshen Fan, Chengping Wen

**Affiliations:** a College of Basic Medical Science, Zhejiang Chinese Medical University; b Department of Rheumatism, The Second Affiliated Hospital of Zhejiang University Medical College; c Department of Rheumatism, Zhejiang Hospital; d Department of Public Health, Medical College, Zhejiang University; e Department of Rheumatism, Zhejiang People's Hospital; f Department of Rheumatism, Sir Run Run Shaw Hospital of Zhejiang University Medical College; g Department of Rheumatism, The First Affiliated Hospital of Zhejiang Chinese Medical University, Hangzhou; h Department of Rheumatism, Chinese Medical Academy Gate Hospital, Beijing; i Department of Rheumatism, The Second Affiliated Hospital of Zhejiang Chinese Medical University, Hangzhou, P.R. China.

**Keywords:** adverse events, double-blind, gout, randomized controlled trial, recurrence of joint swelling and pain, therapeutic effect, traditional Chinese medicine, uric acid

## Abstract

**Trial Design:**

In the double-blind, randomized, controlled trial, we aimed to evaluate the effects of compound tufuling oral liquid (CoTOL) on serum uric acid (sUA) levels and recurrence of acute gouty arthritis in intercritical and chronic gout treatment.

**Methods:**

A total of 210 patients with gout were screened from 8 hospitals to observe the sUA and acute gouty arthritis recurrence rate-reducing effects of CoTOL in intercritical and chronic gout during a 12-week treatment. We treated 139 and 71 patients with CoTOL and the placebo, respectively, and evaluated their sUA levels, acute gouty arthritis recurrence rate, and adverse events at week 0, 6, and 12.

**Results:**

Twenty-five and 12 patients in the treatment and control groups, respectively, had interrupted treatments, whereas 114 and 59 cases, respectively, completed their treatments. At the end of the 12-week treatment, the average decrease in sUA was 74.26 (95% confidence interval [CI]: 56.74–91.77 μmol/L) and 28.81 μmol/L (95% CI: 4.91–52.71 μmol/L) in the treatment and control groups, respectively (*P* = 0.004). The average decrease rate of sUA was 12.76% (95% CI: 9.82%–15.70%) and 4.57% (95% CI: 0.42%–8.71%) in the treatment and control groups, respectively (*P* = 0.004), and the gouty arthritis recurrence rate of the treatment group was lower than that of the control group (from week 6 to 12, 21.93% and 50.88% in the treatment and control group, respectively, *P* < 0.001; from baseline to week 12, 38.5% and 63.16%, respectively, *P* = 0.003). Severe adverse events were not observed in either groups, and fewer leucopenia incidences were observed in the treatment group than those in the control group (3/139 vs. 7/71, respectively, *P* = 0.033).

**Conclusion:**

CoTOL reduced sUA levels and effectively prevented acute arthritis recurrence in intercritical and chronic gout without serious adverse events.

## Abstract 2

Supplemental Digital Content is available in the text

## 1 Introduction

Gout is a metabolic rheumatism characterized by hyperuricemia and recurrent acute gouty arthritis, which is difficult to cure and relapses easily. The etiopathogenesis of gout is related to genetic^[1,2]^ and endocrine factors,^[3]^ purine-rich food intake,^[4]^ and environmental factors,^[5]^ which cause abnormal metabolism, resulting in hyperuricemia. In affected individuals, urate crystals are deposited in the joints, leading to acute gouty arthritis. The prevalence of gout is increasing worldwide^[6]^ and was 1.4% in the UK and Germany from 2000 to 2005,^[7]^ and increased from 6.7 per 1000 inhabitants in 2005 to 9.1 per 1000 inhabitants in 2009 in Italy.^[8]^ A similar trend of increase in gout prevalence has also been observed in China. In Taiwan, the prevalence of a history of gout was found to be 15.2% (25/165) and 4.8% (11/231) in the Aboriginal men and women, respectively, compared with a prevalence rate of 0.3% in non-Aborigines.^[9]^ In 2002, the age-standardized prevalence was 25.3% for hyperuricemia and 0.36% for gout in adults aged 20 to 74 years in Qindao, China.^[10]^

Conventional western medicines used to treat acute gout include anti-inflammatory and analgesic agents (such as colchicine, nonsteroidal anti-inflammatory drugs [NSAIDs], and glucocorticoids),^[11]^ whereas hypouricemic agents (including allopurinol, benzbromarone,^[12]^ and febuxostat^[13]^) are used for chronic gout.^[14]^ Unfortunately, although these drugs show some therapeutic effects, they also cause numerous side effects. For example, colchicine, which inhibits leukocyte migration and lactic acid production to decrease inflammatory responses and urate crystal deposition,^[15]^ may also induce abdominal pain, nausea, vomiting, diarrhea, bone marrow depression with aplasticanemia, agranulocytosis, thrombocytopenia, peripheralneuritis, alopecia, reversible azoospermia, myoneuropathy and myopathy, and rhabdomyolysis.^[16]^ NSAIDs may exacerbate epeptic ulcers,^[17]^ renal failure,^[18]^ hypertension, and cardiovascular disease.^[19]^ Similarly, glucocorticoids may exacerbate peptic ulcers,^[20]^ hypertension, hyperlipidemia, and diabetes,^[21]^ which results in patients with gout discontinuing their treatment. Allopurinol is also limited by adverse events such as hypersensitivity reactions and can even cause death. Therefore, it is of great concern to find effective herb medicines^[22]^ and nonpharmacological treatment methods.^[23]^

In China, numerous traditional Chinese medicines (TCMs) have been used to treat gout for thousands of years. Several studies comparing Chinese herbal decoctions and traditional Western medicines for gout treatment have been reported over the past decade.^[24,25]^ However, the absence of large sample, double-blind, randomized controlled trials (RCTs) affects the reliability and replicability of their conclusions.^[26]^ Therefore, designing and conducting a large-sample double-blind RCT to demonstrate the effectiveness of TCM for gout treatment is of great importance.

Our previous studies showed that CoTOL (also named Quzuo Tongbi recipe), which we developed after years of clinical and experimental research, reduces serum uric acid (sUA) levels in intercritical and chronic gout and alleviates acute arthritis without severe adverse reactions even after long-term use.^[27–30]^ In the present study, we sought to further explore the effect of CoTOL on the sUA levels of patients in the intercritical and chronic gout phase, and the recurrence rate of acute gouty arthritis by conducting a double-blind, multicenter, placebo-controlled, RCT (S1 File).

## 2 Methods

### 2.1 Study design

The trial was designed as a prospective, multicenter, randomized, placebo-controlled, and double-blind trial of CoTOL (Table 1) in patients with intercritical and chronic gout, conducted in China. The trial was approved by the Ethics Committee of Zhejiang Chinese Medical University, and the procedures were conducted in accordance with the Declaration of Helsinki 1975, as revised in 2008.^[31]^ Informed consent was given to every participant, and all participants had provided the written informed consent.

**Table 1:**
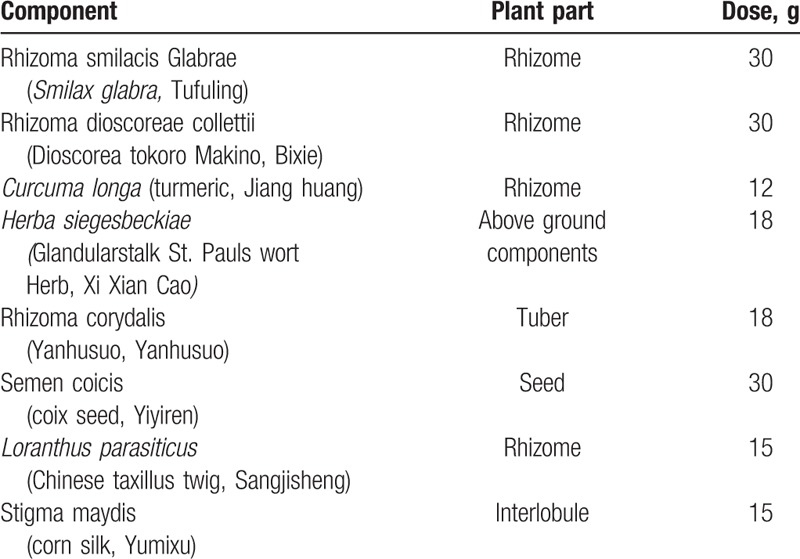
Composition of compound tufuling oral-liquid recipe.

### 2.2 Participants

The inclusion criteria included: male patients, aged 18 to 60 years, with hyperuricemia (blood uric acid [UA] >480 μmol/L), meeting the American College of Rheumatology Classification standard for primary gout revised in 1977 ^[32]^ based on the patient's medical history, without gouty arthritis attack at the time of enrolment, and willing to participate in the trial and sign informed consent certificates. The exclusion criteria included patients: with acute gouty arthritis at the baseline, administered a uricosuric medicine within 2 weeks, with secondary gout, with serum creatinine (Scr) >133 μmol/L or urinary calculi, with serious organ dysfunction, mental illness, or cancer, and body mass index >50 kg/m^2^ or alcoholism. The suspension criteria included: occurrence of serious adverse drug reactions (ADRs) leading to the study team deciding to suspend the patient from the study after adequate evaluation; patients whose sickness worsened after treatment or whose syndrome was influenced by unexpected factors and should be treated as invalid cases; treatment with other gout therapies during the study period, which rendered the participant as an invalid case; and an accumulated treatment interruption of 28 or 21 days at a stretch was regarded as an invalid case. The withdrawal criteria included patients who did not complete the whole treatment course because of serious ADRs or other possible reasons. The cases that were considered withdrawals were managed as follows:

(1) After patients had withdrawn from the study, the researcher coordinators contacted them using every possible method such as personal visits, telephone, or mail to identify their reasons for withdrawing. In addition, the time of their last medication was recorded, and all the possible evaluations were completed.
(2) Patients who withdrew from the study because of allergies or other ADRs were properly treated with other therapies.
(3) All the test data of the withdrawn cases were properly kept as records for use in the total analysis and calculation.

The participants were recruited from June 9, 2012 to May 31, 2013 in 9 hospitals, which were the Rheumatism Institute, Zhejiang Chinese Medical University, Hangzhou, Zhejiang, China; Department of Rheumatism, the Second Affiliated Hospital of Zhejiang University Medical College, Hangzhou, Zhejiang, China; Department of Rheumatism, Zhejiang Hospital, Hangzhou, Zhejiang, China; Department of Rheumatism, Chinese Medical Academy Gate Hospital, Beijing, Beijing, China; Department of Rheumatism, Zhejiang People's Hospital, Hangzhou, Zhejiang, China; Department of Rheumatism, Sir Run Run Shaw Hospital of Zhejiang University Medical College, Hangzhou, Zhejiang, China; Department of Rheumatism, the First Affiliated Hospital of Zhejiang Chinese Medical University, Hangzhou, Zhejiang, China; Department of Public Healthy, Medical College, Zhejiang University, Hangzhou, Zhejiang, China; and Department of Rheumatism, the Second Affiliated Hospital of Zhejiang Chinese Medical University, Hangzhou, Zhejiang, China.

This trial was registered at the Chinese Clinical Trial Registry on June 9, 2012 before participant recruitment and is available on the http://www.chictr.org/ChiCTR-TRC-12002245 website. The authors confirm that all ongoing and related trials for this drug/intervention are registered.

## 3 Randomization and masking

### 3.1 Sample size

The sample size of the measurement data was calculated by using the superiority test: 
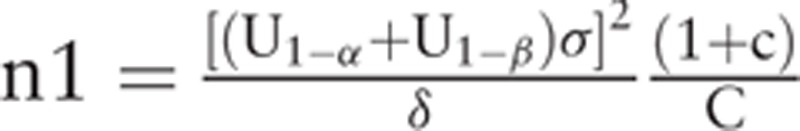
 (where, σ is calculated by pooled standard deviation Sc, δ is equivalent standard, and c is the ratio of 2 sample size); n2 = c n1; c = 2; α = 0.05, β = 0.1, U1-α = 1.64, and U1-β = 1.28. Previous clinical observations showed that the decreased rate of blood UA level after TCM and placebo treatments was 19.37% ± 17.74% and 2.12% ± 4.23%, respectively and, therefore, σ = 14.74 and δ = 6.8. Based on the above formula, the sample size was set at 210 with an expected 20% withdrawal rate based on data from our previous trials.^[27]^ A moderate treatment duration of 12 weeks was chosen out of consideration for patients receiving placebo and for ethical reasons.

### 3.2 Stochastic methods

This study adopted the envelope random method. With each center as a block, the Quality Control Department of the Affiliated Hospitals of Nanjing University of Chinese Medicine used the Statistical Analysis Software (SAS) version 9.2 (SAS Institute, Cary, NC) to generate randomized numbers for each group and patient to determine whether they should be assigned to the treatment or control group.

### 3.3 Masking design

**Figure 1:**
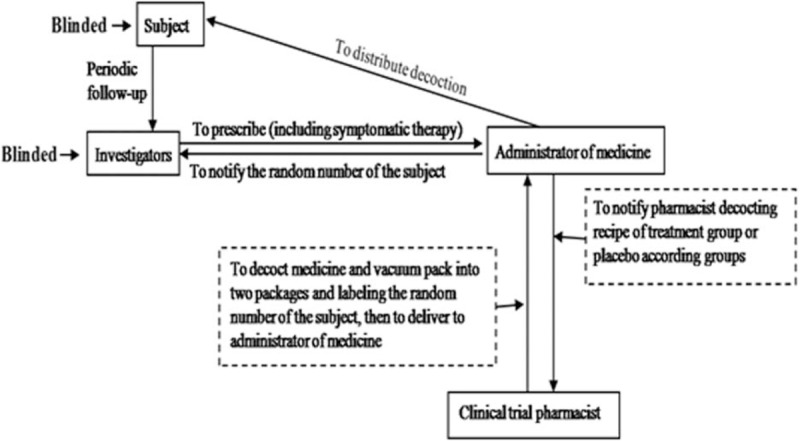
Flowchart of distribution of decoction to implement double-blind trial.

The double-blind method was used to mask the treatments. The researcher coordinators adjusted the basic recipe based on each patient's temporary symptoms and subsequently informed the specially assigned drug management personnel to adjust the procedures afterward. The drug management personnel first obtain each patient's random and group numbers and then informed the pharmacist to decoct their specific treatment recipe or placebo based on the patient's group information (Figs. 1 and Supplementary Fig. 1).

## 4 Interventions

At baseline, every patient was instructed to reduce their purine-rich food intake and to drink >2 L of water a day. The patients randomly assigned to the treatment and placebo groups were administered CoTOL or the placebo (each 2 packs a day), respectively. The age, course of gout, frequency of acute arthritis over 1 year after treatment, joint function, and comorbidities were recorded for each patient.

The treatment recipe was decocted twice by refluxing with water (1:8, w/v) for 1 hour, and then it was vacuum packed into two 250-mL packages. A rapid-performance liquid chromatography identification method was used to authenticate the compound recipe. The control group patients were treated with a placebo consisting of 20 g each of charred millet sprout (Millet sprout, JiaoGuya) and fructus hordei germinatus (Barley sprout, Maiya), 12 g of charred fructus crataegi (Chinese Hawthorn fruit, JiaoShanzha), and 3 g of edible bitter principle, which each have no therapeutic effects on gout. The placebo solution was prepared using the same procedure used for the study drug and vacuum packed into two 250-mL packages, and the color, taste, smell, and physical form of the placebo were the same as those of the treatment recipe.

## 5 Measurements

### 5.1 Efficacy assessments

The primary outcomes were the average decrease and decrease rate of sUA at the end of the 12-week treatment. The average decrease was calculated by using the following formula: C = a − b (where, a and b are the sUAs at baseline, and the end of the 12-week treatment, respectively and C is the decreased sUA value). The decreased rate was calculated by using the formula: R(%) = 100 × (a − b)/a (where, a and b are the sUAs at baseline, and the end of the 12-week treatment, respectively and R is the decreased rate of sUA). The secondary outcome was the decrease in the frequency of recurrent joint swelling or pain. During the 12-week study, fasting venous blood samples were collected in the morning to monitor the biochemical indexes (including sUA). A complete blood count measurement was performed at the baseline as well as at the end of 6 and 12 weeks. The frequency of recurrent joint swelling and pain was recorded at the 6- and 12-week treatment points.

### 5.2 Safety assessments

The adverse events (AEs) including drug-induced liver or renal injury (alanine transaminase [ALT], aspartate transaminase [AST], Scr, urea nitrogen) and hematological parameters (leukocyte, erythrocyte, hemoglobin, glucose (GLU), total cholesterol (TC), high-density lipoprotein cholesterol [HDL-c], and triglyceride [TG]), and vital signs (systolic blood pressure and diastolic blood pressure) were recorded at all scheduled clinic visits.

### 5.3 Quality control

Each patient's clinical information was recorded in a case report form (CRF). Then, 2 data entry staff members entered the data into the electronic CRF on the e-clinical study website (http://www.njecdm.com) administered by the Quality Control Department of the China State Clinical Trial Center of TCM, which was assigned by the State Administration of TCM of the People's Republic of China. The data management was performed by using the web-based clinical trial management platform, ResMan. The data were blindly managed and analyzed by the clinical and statistical staff of the Department of Public Health, Medical College, Zhejiang University.

### 5.4 Statistical analysis

The dataset was first exported from the electronic CRF on the e-clinical study website. The intention-to-treat (ITT) or safety set (SS) analysis included patients who responded to at least 1 follow-up survey, and the per-protocol population set (PPS) analysis included patients who adhered to the treatment protocol. The values are presented as the median (interquartile range) for continuous variable and proportion or ratio for categorical variables. The differences in the decreased value or decreased rate of the sUA between the groups were analyzed using the Wilcoxon signed-rank test. The *χ*^2^ test was used to analyze the difference in the proportion or ratio of categorical variables between the groups. The difference in the recurrence of joint swelling and pain between groups during the treatment course was analyzed by using the Wilcoxon signed-rank test. A 2-tailed *P* value <0.05 was considered statistically significant, and the statistical analyses were performed using the statistical package for the social sciences (SPSS) version 18.0 for Windows (SPSS Inc, Chicago, IL) and the SAS software version 9.2.

## 6 Results

### 6.1 Subjects

**Figure 2:**
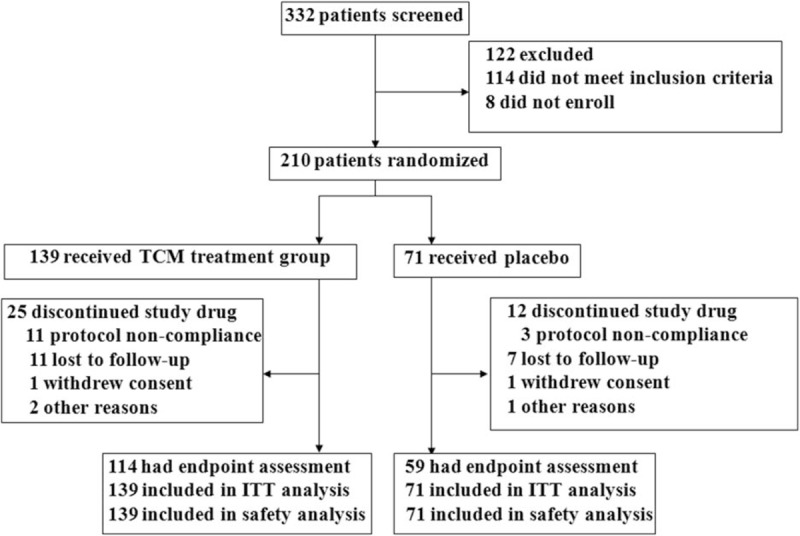
Flow diagram of participant enrollment and treatment. Of the 332 male patients with gout screened for eligibility, 114 did not meet the inclusion criteria while eight did not enroll. Of the 210 patients assessed for eligibility at baseline, 139 and 71 were assigned to the treatment and control groups, and received compound tufuling oral-liquid and the placebo, respectively. During the 12-week follow-up, a total of 25 and 12 patients in the treatment and control groups had interrupted treatment. Thus, 114 patients who underwent the complete course of treatment and had endpoint assessment were included in per-protocol population set analysis and 139 were included in ITT and safety analyses in the treatment group. In addition, 59 patients were included in the per-protocol population set analysis and 71 were included in the ITT and safety analyses in the control group. ITT = intention-to-treat, TCMs = traditional Chinese medicines.

The flow of the participants through the trial is shown in Fig. 2 and Supplementary Fig. 2. Of the 332 male patients with gout who were screened for eligibility, 114 did not meet the inclusion criteria and 8 did not choose to enroll. Of the 210 patients assessed for eligibility at baseline, 139 and 71 were assigned to the treatment and control groups, and received CoTOL and the placebo, respectively. During the 12-week follow-up, a total of 25 and 12 patients in the treatment and control groups had interrupted treatment. Thus, 114 patients who underwent the complete course of treatment and had endpoint assessment were included in the PPS analysis and 139 were included in the ITT and safety analyses in the treatment group. Furthermore, 59 patients were included in PPS analysis and 71 were included in ITT and safety analyses in the control group.

### 6.2 Baseline characteristics

Table 2 shows the baseline characteristics of the included patients. The demographical and clinical characteristics were well balanced between both groups. The median age was 46 and 49 years in the treatment and control groups, respectively. The median course of disease was 7 and 5 years in the treatment and control groups, respectively. The median frequency of acute arthritis in 1 year was 3 and 4 in the treatment and control groups, respectively. The median sUA levels were 550 and 530 μmol/L in the treatment and control groups, respectively.

**Table 2:**
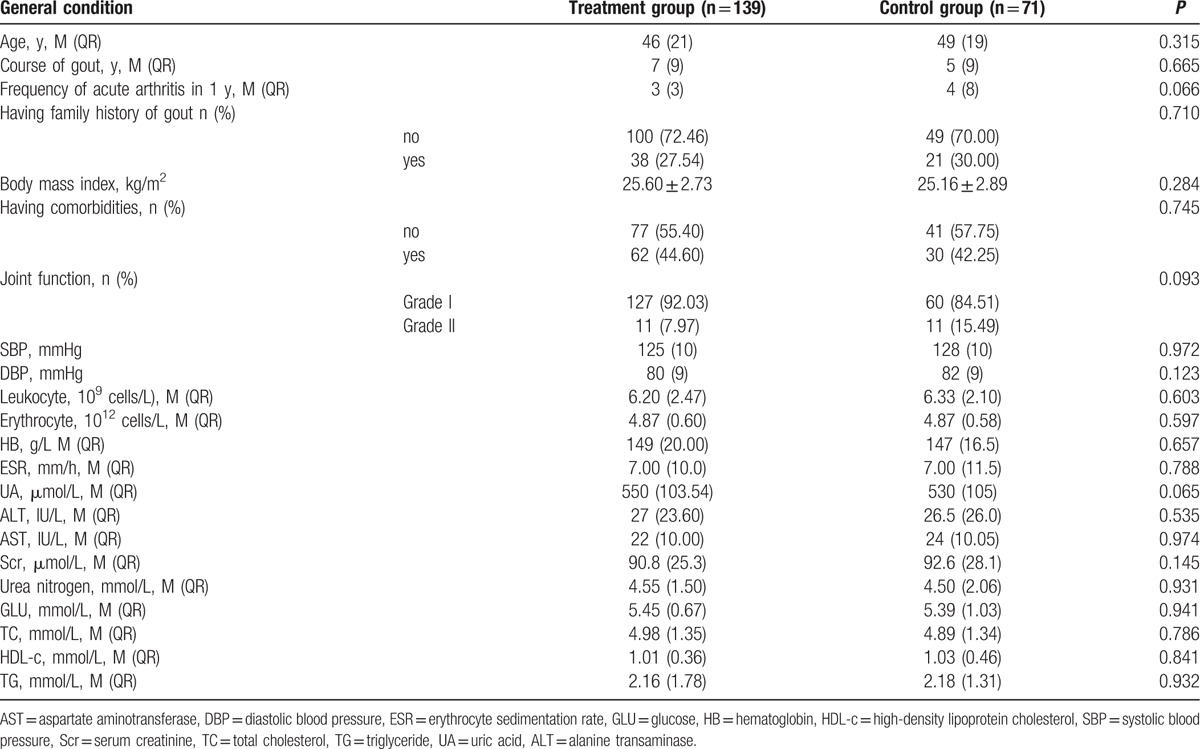
Patient baseline characteristics.

### 6.3 Efficacy

**Figure 3:**
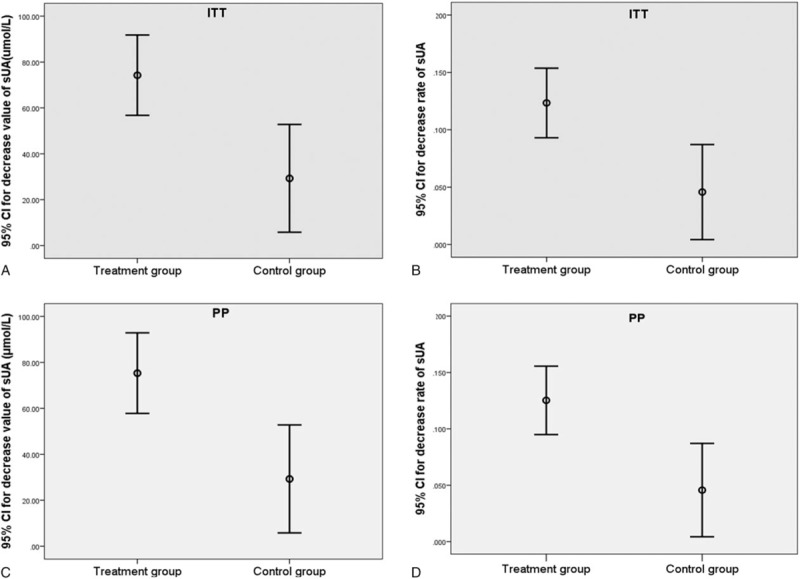
sUA reduction by compound tufuling oral-liquid. (A) ITT analysis of decreased sUA after 12-week treatment. The difference between both groups was statistically significant (*z* = −2.87*, P* = 0.004, Wilcoxon rank sum test). (B) Intention-to-treat analysis of decreased rate of sUA after 12-week treatment. The difference between the 2 groups was statistically significant (*z* = −2.86*, P* = 0.004, Wilcoxon rank sum test). (C) PPS analysis of decrease sUA after 12-week treatment. The difference between the 2 groups was statistically significant (*z* = −2.956*, P* = 0.003, Wilcoxon rank sum test). (D) PPS analysis of decrease rate of sUA after 12-week treatment. The difference between the 2 groups was statistically significant (*z* = −2.954*, P* = 0.003, Wilcoxon rank sum test). CI = confidence interval, PPS = per-protocol population set, sUA = serum uric acid.

Using an ITT analysis, we found that at the end of week 12, the average decrease in the sUA was 74.26 μmol/L (95% confidence interval [CI]: 56.74–91.77 μmol/L) in treatment group versus 28.81 μmol/L (95% CI: 4.91–52.71 μmol/L) in the control group (*z* = −2.87*, P* = 0.004, Wilcoxon rank sum test). Moreover, the average decreased rate of the sUA was 12.76% (95% CI: 9.82%–15.70%) in the treatment group versus 4.57% (95% CI: 0.42%–8.71%) in the control group (*z* = −2.86*, P* = 0.004, Wilcoxon rank sum test, Fig. 3 A and B and Supplementary Fig. 3A and B).

The PPS analysis also showed similar results and the average decrease in the sUA at the end of week 12 was 75.34 μmol/L (95% CI: 57.80–92.88 μmol/L) in treatment group versus 28.81 μmol/L (95% CI: 4.91–52.71 μmol/L) in the control group (*z* = −2.956, *P* = 0.003, Wilcoxon rank sum test). Furthermore, the average decrease rate of sUA was 12.96% (95% CI: 10.01%–15.90%) in the treatment group versus 4.57% (95% CI: 0.42%–8.71%) in the control group (*z* = −2.954*, P* = 0.003, Wilcoxon rank sum test, Fig. 3C and D and Supplementary Fig. 3C and D).

**Figure 4:**
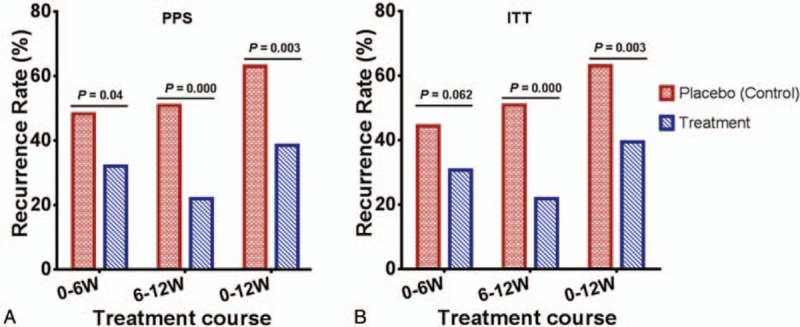
Prevention of gouty arthritis recurrence by compound tufuling oral-liquid. (A) PPS analysis (Pearson *χ*^2^ test): from baseline to end of week 6, 32.14% and 48.28% in treatment and control group, respectively, *P* = 0.04; from end of week 6 to end of week 12, 22.02% and 50.88% in treatment and control group, respectively, *P* < 0.001; and from baseline to end of week 12, 38.53% and 63.16%in treatment and control group, *P* = 0.003. (B) ITT analysis (Pearson *χ*^2^ test): from baseline to end of week 6, 30.77% and 44.44% in treatment and control group, respectively, *P* = 0.062; from end of week 6 to end of week 12, 21.93% and 50.88% in treatment and control group, respectively, *P* < 0.001; and from baseline to end of week 12, 39.50% and 63.16% in treatment and control group, respectively, *P* = 0.003. ITT = intention-to-treat, PPS = per-protocol population set.

The PPS analysis (Pearson *χ*^2^ test) revealed that the recurrence rate of gouty arthritis (joint swelling or pain) in the treatment group was lower than that in the control group. The specific values were: from baseline to the end of week 6, 32.14% and 48.28% in the treatment and control groups, respectively, *P* = 0.04; from the end of week 6 to the end of week 12, 22.02% and 50.88% in the treatment and control groups, respectively, *P* < 0.001; and from baseline to the end of week 12, 38.53% and 63.16% in the treatment and control groups, respectively, *P* = 0.003, Fig. 4 A and Supplementary Fig. 4A. The ITT analysis (Pearson *χ*^2^ test) revealed that the recurrence rate of gouty arthritis in the treatment group was lower than that in the control group. The specific values were: from baseline to the end of week 6, 30.77% and 44.44% in the treatment and control groups, respectively, *P* = 0.062; from the end of 6 to the end of week 12, 21.93% in the treatment and control groups, respectively; *P* = 0.000; and from baseline to the end of week 12, 39.50% and 63.16% in the treatment and control groups, respectively, *P* = 0.003, Fig. 4B and Supplementary Fig. 4B.

### 6.4 Safety

At week 0 and the end of week 12 after treatment, several laboratory indices including the blood cell count, alanine transaminase, aspartate transaminase, GLU), Scr, TC, TG, HDL-c, and occult blood stool were measured. Except for the leukocyte count, no obvious abnormalities or indications of worsening symptoms were observed in the 2 groups during the treatment. Fewer cases of leukopenia were observed in the treatment group than in the control group (3/139 and 7/71 in treatment and control group, respectively, *P* = 0.033, Table 3).

**Table 3:**
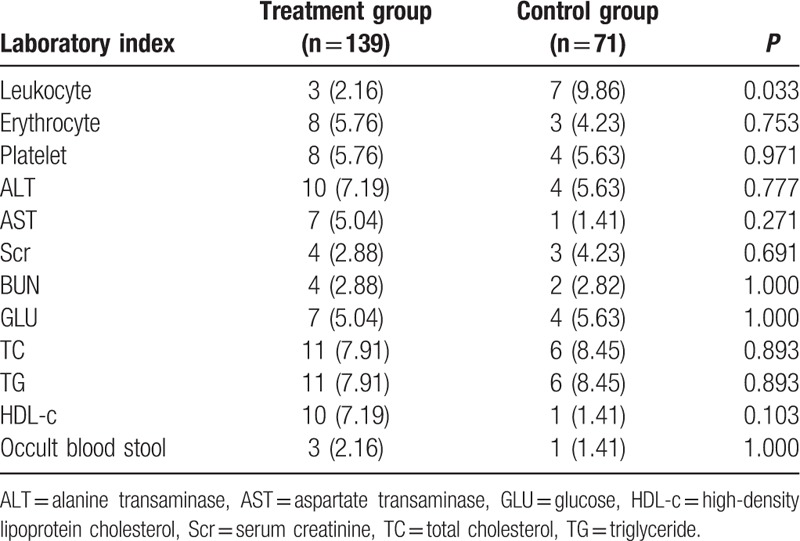
Incidence rates of abnormal laboratory indices (M [QR]).

## 7 Discussion

Gout is a metabolic rheumatic disease caused by long-term abnormal purine metabolism. The developmental process of primary gout can be divided roughly into the hyperuricemia (without gouty symptoms), acute gout, and chronic gout (including the development of chronic gouty arthritis and gouty stones) phases. The prevalence of gout is increasing worldwide ^[6]^ and conventional medicines including colchicine, NSAIDS, allopurinol, and benzbromarone have some therapeutic effects on gout,^[33]^ but they also have numerous side effects. For example, allopurinol is limited by an AE profile that includes hepatic, gastrointestinal, renal, hematological, and skin toxicities that occur in approximately 20% of patients. In addition, this drug may cause the death of 2% to 4% of patients with hypersensitivity reactions. Notably, patients tend to show low compliance and tolerance to these drugs, thereby limiting their long-term effectiveness. Consequently, there is an urgent need for the identification of new strategies to control acute gouty arthritis and the development of new drugs to lower sUA levels.^[34,35]^

Based on previous studies,^[27–30]^ we adopted a double-blind, placebo-controlled, multicenter, randomized trial design to determine the effect of CoTOL. Our results indicate that the decrease in the sUA level of the treatment group was significantly higher than that of the control group. Furthermore, the frequency of recurrent joint swelling or pain in the treatment group was obviously lower than that in the control group, whereas the number of patients with an exacerbated white blood cell count was lower in the treatment group than it was in the control group. In addition, severe AEs did not occur in either group. Two and 8 patients in the treatment and control groups, respectively, experienced mild AEs. Therefore, our results indicate that CoTOL reduced the sUA levels of patients in the intercritical and chronic gout phases and prevented the recurrence of acute gouty arthritis with a better efficiency than the placebo did.

CoTOL consists mainly of Rhizoma smilacis Glabrae (*Smilax glabra,* Tufuling), Rhizoma dioscoreae collettii (*Dioscoreatokoro* Makino, Bixie), *Curcuma longa* (turmeric, Jianghuang), Herba siegesbeckiae (Glandularstalk St.Paul's Wort herb, Xixiancao), and Rhizoma corydalis (Yanhusuo, Yanhusuo), Semen coicis (coix seed, Yiyiren), *Loranthus parasiticus* (Chinese taxillus twig, Sangjisheng), and Stigma maydis (corn silk, Yumixu). Several pharmacological studies have shown that these herbs may have positive effects on gout. For example, Rhizomasmilacis glabrae has anti-inflammatory and analgesic actions.^[36]^ As a flavonoid compound isolated from Rhizoma smilacis Glabrae, astilbin has anti-inflammatory effects.^[37]^ In addition, astilbinis effective in preventing hyperuricemia and nephropathy by increasing the urinary UA level and fractional excretion of urate, preventing renal damage by the expression of transforming growth factor-β1 and connective tissue growth factor, and inhibiting the formation of monosodium urate as well as the production of prostaglandin E_2_ and interleukin-1.^[38]^ These findings provide strong evidence supporting the use of astilbin as a safe and promising lead compound for the prevention of hyperuricemia and gouty nephropathy. *D tokoro Makino* and its saponin ingredients, which have been shown to decrease foot swelling in mice and rats and heighten the pain threshold of mice, have positive effects on gouty arthritis induced by sodium urate by reducing sUA.^[39,40]^ Herba siegesbeckiae has anti-inflammatory and analgesic effects.^[41–43]^ Rhizoma corydalis has analgesic effects and is effective for treating gastric ulcer.^[44,45]^*C longa*, which contains 2 classes of secondary metabolites namely curcuminoids and essential oils, possesses effective inhibitory activity against xanthine oxidase^[46]^ as well as anti-inflammatory and antiarthritic effects.^[47–49]^ Because of its strong antioxidant capacity and recovery of cisplatin-induced nephrotoxicity, *L parasiticus* exhibits potent anti-inflammatory and antinephrotoxic activities,^[50–52]^ which may be beneficial for reducing the recurrence of gouty arthritis and preventing urate-induced renal damage. Semen coicis shows analgesic and anti-inflammatory properties.^[53,54]^ Stigma maydis (corn silk), which contain saponin, alkaloid, flavones, volatile oil, mucilage, Vitamins B, C, and K, Silicon, increases the urinary output and the percentage the passage of urinary stones through the urinary tracts without decomposed stones, lower the total amount of acid in the urine,^[55,56]^ and inhibit xanthine oxidase,^[57]^ Therefore, Stigma maydis play an active role in treating gout by inhibiting the production of sUA, promoting the excretion of UA, and preventing the formation of kidney stones.

Therefore, we hypothesized that CoTOL might lower sUA and reduce the recurrence of gouty arthritis. The present study clearly demonstrates that the median sUA levels of patients treated with CoTOL were significantly decreased compared to those of the patients treated with placebo after 12 weeks, whereas the recurrence rate of joint swelling or pain in the treatment group was lower than that in the placebo control group. Moreover, severe AEs did not occur in either group. Only 2 participants experienced mild AEs and three manifested leukopenia in the treatment group, which was unrelated to CoTOL.

## 8 Conclusions

In conclusion, our findings suggest that CoTOL has significant therapeutic potential not only by reducing the sUA level but also by decreasing the frequency of recurrent joint swelling or pain without obvious AEs. The study has limitations including the fact that the observation period was limited to 12 weeks, and no positive control was used. Future studies should involve a trial with a longer treatment course and a positive control drug to provide further evidence of the efficacy of CoTOL in treating gout. Furthermore, its mechanisms of action also need to be investigated.

## Supplementary Material

Supplemental Digital Content
