# Supplementary material for: Hypouricemic and arthritis relapse-reducing effects of compound tufuling oral-liquid in intercritical and chronic gout: A double-blind, placebo-controlled, multicenter randomized trial
Source: Medicine (Baltimore). 2017 Mar 24;96(11):e6315. doi: 10.1097/MD.0000000000006315 (PMC5369899; doi:10.1097/MD.0000000000006315)
Supplement: Supplemental Digital Content [file medi-96-e6315-s001.doc]

**S1 File.** Trial study protocol

**Project Initiation:** the Eleventh Five-Year Scientific Support Plan of the [Ministry of Science and Technology](app:ds:Ministry of Science and Technology)

**Project Title:** Hypouricemic and Arthritis Relapse-reducing Effects of Compound Tufuling Oral-Liquid on Intercritical and Chronic Gout: A Double-blind, Placebo-controlled, Multicenter Randomized Trial

**Project Number:** 2007BAI20B06

**Project Director:** Wen Chengping

**Undertaking Institute:** Zhejiang University of Chinese Medicine

**Contact Person:** Wen Chengping

**Contact Number:** +86 13906514781

**Study Period:** Jun. 2012－Dec. 2014

# I. Study Purpose

## To evaluate the clinical effect of Compound Tufuling Oral-liquid on the reduction of serum Uric Acid (UA) level and recurrence of acute gouty arthritis in intercritical and chronic gout.

# II. Design Methods and Principles

## (I) Study Design: randomized block design, double-blind and placebo parallel control, multi-center clinical study, superiority test

## (II) Sample Size: calculation formula of the sample size of measurement data by superiority test ^[23]^:
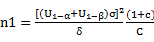
 (σ calculated by polled standard deviation Sc, δ is equivalent standard, c is the ratio of two sample size)

## n2 = c*n1; c=2; α=0.05, β=0.1, U_1-α_=1.64, U_1-β_=1.28; previous clinical observation showed that the decrease rate of blood UA level after TCM treatment and placebo treatment was respectively (19.37±17.74)% and (2.12±4.23)%, so σ=14.74; δ=6.8

## Based on the above formula, we calculated the sample size of the treatment group and placebo group being respectively 120 and 60. Considering a 20% withdraw rate, the total sample size was decided 216.

## (III) Stochastic Methods: This study adopted envelope random method. With each center as a block, we use SAS software version 9.2 (SAS Institute, Cary, NC, USA) to generate randomized number for each group and each patient to decide whether they should belong to the treatment group or controlled group.

## (IV) Control**:** TCM placebo controlled

## (V) Masking Design**:** double-blind method. The researchers adjust the basic recipe according to every patient’s temporary symptoms and inform the specially-assigned drug management personnel for afterwards procedures. The drug management personnel should firstly obtain each patient’s random number and group number, and then inform the pharmacist to decoct his/her treatment recipe or placebo as the patient’s group shows. The decocted herb solutions would be vacuum packed to be handed into patient’s hands.

# IV. Study Population

## (I) Source of Cases: 216 outpatients and inpatients of intercritical gout chosen from six hospitals, namely, the First Affiliated Hospital of Zhejiang University of Chinese Medicine, the Second Affiliated Hospital of Zhejiang University of Chinese Medicine, the third Affiliated Hospital of Zhejiang University of Chinese Medicine, the Second Affiliated Hospital of Zhejiang University School of Medicine, Zhejiang Hospital, Guang’anmen Hospital of the China Academy of Chinese Medical Science.

## (II) Diagnosis Criteria

1．Western Medicine Diagnosis Criteria: the criteria for the classification of primary gout revised by the American College of Rheumatology in 1977^[24]^:

1977 Criteria for the Classification of Acute Arthritis of Primary Gout

| 1. Specific urate crystals in joint fluid or 2. Urate crystals in tophi proven by chemical tests or polarized light microscopy or 3. 6 out of the following 12 manifestations:    1. More than one attack of acute arthritis    2. Maximum inflammation developed within 1 day    3. Monoarthritis attack    4. Redness observed over joints    5. First metatarsophalangeal joint painful or swollen    6. Unilateral first metatarsophalangeal joint attack    7. Unilateral tarsal joint attack    8. Tophus (proven or suspected)    9. Hyperuricemia    10. Asymmetric swelling within a joint on x ray    11. Subcortical cysts without erosions on x ray    12. Joint fluid culture negative for organisms during attack |
| --- |

Intercritical gout: gout lasted for many years with sustained high density of hyperuricemia, mainly diagnosed by the past attack of acute arthritis of primary gout and hyperuricemia

2．Chinese Medicine Diagnosis Criteria:

We divide gout into two phases, acute phase and intercritical phase, based on *the Criteria for the Classification of Primary Gout revised by the American College of Rheumatology* in 1977 and *the Guidelines for the Diagnosis and Treatment of Primary Gout revised by the Rheumatism Branch of Chinese Medical Association* in 2003.

According to the *Curative Standard for TCM Diagnosis revised by the State Administration of TCM*, combined with our clinical experience, we worked out a TCM diagnosis criteria for intercritical gout as follows:

TCM disease name: Bi Syndrome (intercritical)

TCM syndrome: spleen deficiency and dampness-phlegm obstruction

Syndrome differentiation standard: gout lasted for many years with sustained high density of hyperuricemia and such symptoms as dull pain in joints, fatigue, abdominal distension, anorexia, loose stool, red tongue with white greasy fur or powder greasy fur, and slippery pulse.

## (III) Inclusion Criteria:

(1) In intercritical phase of primary gout;

(2) Diagnosed with spleen deficiency and dampness-phlegm obstruction syndrome;

(3) Blood Uric Acid≥480μmol/L;

(4) Males between 18 to 68 years old;

(5) Voluntarily joined the clinical study and agree to sign an informed consent certificate.

## (IV) Exclusion Criteria:

1. In acute phase of gout or with tophi
2. Took medicines to reduce UA levels within 2 weeks before the study
3. Secondary hyperuricemia or gout, such as congenital metabolic diseases, myeloproliferative disorders, late stage of hypertension disease, hyperuricemia and kidney failure caused by diabetic ketoacidosis, cancer chemotherapy and/or radiotherapy
4. Arthropathies caused by diseases like [rheumatoid arthritis](app:ds:rheumatoid arthritis), purulent arthritis, traumatic arthritis, psoriatic arthritis, pseudogout, systemic lupus erithematosus
5. Had a serum creatinine level ≥133μmol/L or urinary calculi
6. Had serious organ dysfunctions, mental illness or cancer
7. Had a body mass index ≥50kg/m^2^, or were alcoholic
8. Had low tolerance to the drugs of this study
9. Other inadequacy judged by the study team

## (V) Suspension Criteria

(1) Serious adverse drug reactions (ADRs) occur and the study team decide to suspend the study after adequate evaluation.

(2) Patients whose sickness get worse after treatment or whose syndrome been influenced by unexpected factors should be treated as invalid cases. Their treatment should be suspended.

(3) Patients treated with other gout therapies during the study period should be counted as invalid cases.

(4) Patients with accumulated treatment interruption period of 28 days or 21 days for once should be regarded as invalid cases.

## (VI) Withdraw Criteria

1．Withdraw criteria: patients who didn’t finish the whole treatment course because of serious ADRs or other possible reasons should be regarded as withdraw cases

2．Management of withdraw cases

(1) After patients withdrew from the study, researchers should contact them in every possible way, such as by visit, telephone or mail, to ask their reasons of withdraw, record the time of their last medication and finish all the possible evaluation items.

(2) Patients who quitted the study because of allergy or other ADRs should be properly treated by other therapies.

(3) All the test data of withdraw cases should be well kept as records for total analysis and calculation.

## (VII) Expulsion Criteria

1．Patients who did not match the inclusion criteria

2．Patients who didn’t take medicine as required during the study

3．Patients who were assigned no number in randomization

Before the statistical analysis, statisticians and the main researchers should decide excluded cases with discussion.

# V. Treatment Plan

## (I) Therapy

1. Controlled therapy: TCM placebo

2. Treatment therapy: Compound Tufuling Oral-liquid therapy in combination with temporary symptomatic therapy

Every herb of the treatment therapy was purchased from the same batch and processed into decoction pieces. We carried out quality tests for processed decoction pieces, including their region of growth, shape and properties, species, fingerprinting and impurities. Quality test reports of the content of each decoction pieces were available, which were in accordance with the State Pharmacopoeia Standard. Samples of all the herbs were kept for future inspection.

3. Management of therapies

Processed herbs were allocated to six treatment centers according to their case number. There was a specially-assigned management person in each center to be in charge of the registration, store, and management of herbs. There was another assistant to distribute decoction pieces according to doctor’s prescription. Then each center had a pharmacist specially assigned to decoct each patient’s recipe following the same decoction method and procedure. Finally, the decocted solutions were delivered to the patients.

## (II) Basic Treatment

1．Reminded patients to keep fit and maintain normal body weight

2．Proper Diet:

(1) Low purine diet. Daily purine intake should be controlled fewer than 100 ~ 150mg. Protein intake should also be properly limited. Alkaline food was preferred, such as vegetables, potatoes, sweet potatoes, milk, and oranges. Alcohol, meat soup, animal giblets and bone marrow, seafood, clams and crabs were prohibited. Square meal and large sum of soybean food should be avoided. We recommended a healthy meal with rice, vegetables, milk, eggs and fruits.

(2) Patients were required to drink 2500 ~ 3000ml liquid every day and a little more water before sleep to ensure a daily urine volume of 2000ml approximately. Beverages like plain water, tea, mineral water, soft drinks and fruit juice were good to them but strong tea, coffee and cocoa should not be drunk. Patients were also asked to keep exercise habit.

3．Urine Alkalifying: take 1g sodium bicarbonate three times a day to keep the PH value of urine around 6.5.

## (III) Medication

1. Controlled therapy: TCM placebo consisted of 20 g each of charred millet sprout (JiaoGuya) and fructus hordei germinatus (Maiya), 12 g of charred fructus crataegi (JiaoShanzha), and 3g of edible bitter principle) .

2．Treatment therapy**:** Compound Tufuling Oral-liquid consisted of Rhizoma smilacis Glabrae (Tufuling) 30g, Rhizoma dioscoreaecollettii (Bixie) 30g, Curcuma longa (Jianghuang) 12g, Herba siegesbeckiae (Xixiancao) 18g, Rhizoma corydalis (Yanhusuo)18g, Semen coicis (Yiyiren) 30g, Loranthus parasiticus (Sangjisheng)15g, Stigma maydis (Yumixu)15g.

All the above recipes were decocted by the pharmacy department of each center following unified procedures: firstly immersed in 700ml water for 30 minutes, and then decocted in automatic tisanes machines on lowfire for 40 minutes. The decocted solution was packed by automatic liquid packaging machines into two vacuum packages, each with 150ml solution ready to dose patients, one package each time and twice a day after meal. The quality guarantee period of decocted herb solution packages was 7 days under normal temperature and 14 days below 4 degrees centigrade, which allowed patients to pick up them every 1 or 2 weeks.

3．Drug Combination

No other UA lowering drugs were used other than our treatment therapy, either western medicine or TCM.

If severe pain occurred to patients with acute arthritis of primary gout during the treatment course, 15mg Meloxicam capsules per time per day would be prescribed to relief the pain. A reduction or stop of this prescription should be considered after pain relief.

For patients accompanied with diseases like hyperlipidemia (blood triglycerides>1.7mmol/l and/or total cholesterol>5.72mmol/l and/or high density lipoprotein cholesterin<1.04mmol/l), diabetes mellitus (fasting vein plasma glucose≥7.0mmol/l or optional vein plasma glucose≥11.1mmol/l or vein plasma glucose≥11.1mmol/l 2h after OGTT), hypertension (blood pressure>140/90mmHg), and [coronary](app:ds:coronary) [heart](app:ds:heart) [disease](app:ds:disease) (with onset angina pectoris, ischemic electrocardiographic changes at rest, or positive electrocardiogram test result), researchers should help them with diet adjustment or conventional western medicine treatment when necessary.

## (IV) Course of treatment**:** 12 weeks

## (V) Contraindication: Patients who had acute or chronic kidney failure or cardiac insufficiency and who were allergic to the treatment therapy or had obvious gastrointestinal reactions after dosages should be prohibited to take the treatment therapy.

# VI. Investigation Items

## (I) Normal Recording Items

Patients’ name, the initial letters of its phonetic symbol, outpatient and inpatient treatment history, marriage state, onset process, date of definite diagnosis, date joining the study

## (II) Investigation Items

1. Biological Indexes

(1) Demographic characteristics: gender, age, height, weight

(2) Vital signs: body temperature, resting heart rate, breathe, blood pressure after 5 minutes of rest including systolic and diastolic pressure

2. Curative Indexes

(1) Main Index:

Decreased rate of blood UA level at the 12^th^ weekend compared with the baseline(
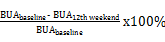
)

(2) Minor Index:

Recurrence rate of joint swelling or pain

3. Influencing Factors

Chronic arthritis, function of joint, course of disease, attack frequency in the last year

Renal function: routine urinalysis, renal function test

Associating disease: medical history of overweight, type 2 diabetes, hyperlipidemia, hypertension, atherosclerosis, or [coronary](app:ds:coronary) [heart](app:ds:heart) [disease](app:ds:disease)

Diagnostic indexes of associating diseases: body mass index(BMI)=weight(kg)/hight^2^(m^2^), Glu, TC, TG, HDL-C, BP, electrocardiogram

Recent medicinal history

Inducing factors: local damages of the joints such as food sprain, long-time walking with tight shoes, catching cold, alcohol drinking

4. Safety Indexes

Routine analysis of blood/urine/stool, occult blood test of stool, liver function test (ALT, AST), renal function test (BUN, Scr)

Adverse effects: gastrointestinal reaction, cutaneous eruption

Safety evaluations

5. Evaluation Indexes of the Study

Combined medication, medication compliance, withdraw rate

## (III) Observation Point

1．Lab indexes monitoring: routine analysis of blood/urine/stool, occult blood test of stool, serum creatinine test, renal function test, blood UA test, blood fat test and blood sugar test at the beginning, the 6^th^ weekend and the 12^th^ weekend of the study; electrocardiogram and B ultrasonic examination of urinary system as necessary

2．Recurrence of joint swelling or pain: recorded at the 6^th^ weekend and the 12^th^ weekend of the study

3．Others: normal information, family history of primary gout, drug allergy history, other disease history and medication before the study recorded at the beginning; vital signs, systematic physical examinations at the beginning, the 6^th^ weekend and the 12^th^ weekend of the study; ADRs observed and recorded at all times

# VII. Curative Effect Judgment

1．Judgment of UA level decrease:

Decreased rate of blood UA level at the 12^th^ weekend compared with the baseline (
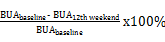
 )

2．Judgment prevention effect of recurrent joint swelling or pain:

Recurrence rate of joint swelling or pain within 12 weeks of study

# VIII. ADRs Observation and Analysis

## (I) Possible ADRs: gastrointestinal reaction

## (II) Record of ADRs

During the study process, researchers should record all the ADRs truthfully in detail, such as the clinical manifestations, occurrence time, severity, duration, actions taken and prognosis, whether they were caused by the study or not. Combined medication should also be recorded carefully in order to estimate the relationship between AE occurrence and treatment therapy.

## (III) Severity Classification of ADRs

Mild: tolerable and do not influence patients’ treatment or health thus do not need any action to be taken

Moderate: hard to tolerate and have impact on patients’ health thus need treatment suspension or other proper arrangement

Severe: endanger patients’ lives and may lead to death or disability thus need immediate treatment suspension and emergency treatment

## (IV) Judgment of the Relevance between ADRs and Treatment Therapy

| Judgment Indexes | Judgment Result | | | | |
| --- | --- | --- | --- | --- | --- |
|  | No doubt | Most likely | Possible | Suspicious | Impossible |
| 1. Whether there is precedence relationship between the drug taking time and the occurrence of ADRs | + | + | + | + | + |
| 1. Suspected ADRs comply with known ADRs of the drug used | + | + | + | - | - |
| 1. Suspected ADRs can be explained by patients’ pathological condition, combined medication, combined therapy or past therapy | - | - | ± | ± | + |
| 1. Whether suspected ADRs relieve or disappear after reduction or withdrawal of drugs | + | + | ± | ± | - |
| 1. The same ADRs appear after reuse of the same drug | + | ？ | ？ | ？ | - |

(V) Management for serious AE

Serious AE is the situation happened in clinical study that need to hospitalization or more time for hospitalization or permanent/severe disability or life threatering or death or congenital malformation.

The researchers must adopt effectively therapeutic measures in hand for the patients who appeared the serious AE, and fill in *Form for Serious AE* and hand in Pharmaceutical Supervisory and Administrative Department , the application unit, unit responsible for clinical research , Medical Ethics Committee and the Administrative Department of Public Health in 24 hours.

**Ⅸ.** **Quality Control (QC) and Quality Assurance(QA) System**

(Ⅰ) Analysis for influencing factor of the research

Diagnosis bias: preliminary screening and diagnosis by the local CDC and clinical experts, and then diagnosis and affirm by expert. So the researchers can control the subjects in the study.

Informational bias in clinical observation: reduce the bias by special personnel in charge, assemble for training, strictly execute SOP for all posts personnel.

Informational bias in data management: reduce the bias by 4 Level monitoring system , relevant SOP and data management system.

Mixed bias: adopte the same foundation treatment for treatment group and control group, so avoide mixed bias caused by complex intervention measures.

(Ⅱ) Collection requirements for examination indicators (physicochemical examine ,scale, etc.): cryopreservation for blood sample, the same batch were detected by the same people in the same laboratory.

(Ⅲ) Training for researchers: take charge by the specialist from research center, including research process, implementation, informed consent implementation, concrete operations for clinical observation index, standard measures for check clinical sign, fillout medical record and CRF table, AE observation and report.

Training carried out in accordance with SOP strictly.

(Ⅳ) Improved patients’ compliance: subjects’ coordination is very important. The researchers must make them clearly understand the significance of the study, and ask them actively cooperate with the treatment and observation. The researchers must explain all the details, including objective, process, deadline, examination, expected benefit and potential risk, and maybe distributed to different groups. The patients’ list and all their personal information are keep in secret. The researchers must keep close contact with the subjects. There is impeccable emergency treatment.

(Ⅴ) Quality control and quality assurance system: take specially-assigned person for archives keeping, data analysis, instrument detection.Ensure the quality by legislate rules and regulations, organization measure and supervision and management system. Monitor examine the implementation anytime, verify all the observations and finding in the clinical research and make sure that all the conclusions are derived from initial data.

**Ⅹ. Data Management**

(Ⅰ) Source file definition

Source file is the first-hand data in the clinical trial. In this clinical research, source file refers to medical record , information consent form, physicochemical examine, drug management files and the file of quality inspect.

(Ⅱ) Data record and report

All the observations and the examine results must record in source file timely, precisely, entirety, norm and truly.

Modification for source file and data must have abundant evidence, or else take the first judgment ( record ) for truth.

Data report adopt webbased electronic CRF and data management system.

Data entry way: independent and duplicate way.

(Ⅲ) Rules for data inspection

Clinical research organization must offer convenient when the monitors in SDW, and consult in the provision places.

Data inspection measure: monitors land data monitoring interlinkage of electronic CRF and data management system for affirmed the different data, and check all the data in electronic CRF in accordance with the source data.

Inquiry for source data must follow the hospital medical file and drugs clinical research institutions archives file.

(Ⅳ) Provisions for the data question and answer

Issue: questions come from logical check and SDV are downloaded by CRC- inputers periodic in form of DCF.

Answer: CRC- inputers take the DCF to the researchers who would modificated the data, and the DCF preserve as source file.

Update online: CRC- inputers update the data on CDF online.

Resolution: Monitors take SDV for DCF, and check the authenticity and rationality. The data administrators check the modificated data, *confirmed* or *new question* when there is still question or find new question, and the question viewed as resolved.

**Ⅺ. Statistical analysis plan**

(Ⅰ) Analysis data set

1. ITT: set of qualified cases and evaluable deciduous cases, but not include excluded cases.

2. PP: including the cases fit inclusion criteria and complete treatment, that is cases that accorded with the research, will compliance, no drug combination and whole CRF.

3. Safety evaluation data sets: patients treated and recorded safety evaluation once at least.

(Ⅱ) Statistical analysis content

Statistical analysis plan is written by the statisticians, including data management, statistical method and analysis content. Finish statistical report according to the statistical results, including experimental units and statistical report of summarized data.

Main analysis:

1. Cases distribution of two groups: comparison of total expulsion rate and expulsion rate caused by AE.
2. Comparability analysis: measure the comparability of two groups by comparison of demographic data and others baseline.
3. Compliance analysis: compare the patients of two groups whether take medicine on time, on proper dosage or not, and take the medicine that forbidden in the research.
4. Effectiveness analysis: take PP and ITT for main indicators and overall indicators. Because of the multicenter clinical trials, we take centrality into account: non-inferiority analysis of two groups for overall indicators.

5. Analysis of factor affect curative effect: such as age, sex, chalkstone or not, chornic arthritis, articular malformation, kidney stone, frequency onset in the last year, renal function, associated disease, recent medication history and precipitating factor, etc..

6. Safety analysis: table the AE and adverse reaction according to the correlation requirements of adverse reaction, including AE cases, cases of normal turn abnormal before and after test and the ratio. Take chi-square test for AE statistic analysis.

(Ⅲ) Statistical analysis method

1 Statistical describe method

① Whether conform to the normal distribution or not: if not, converted the data to the normal distribution and then take parametric statistics, or take nonparametric statistics directly.

② Outliers or not: take statistics and professional analysis, and then decided to accept or reject.

③ Missing data or not: when happened, from the statistics and professional perspectives, choose to expulse or convert.

④ Expulsion rate: 20%.

⑤ Descriptive statistical analysis: Average ± standard deviation or M(Q_R_) for measurement data, and ratio or constituent ratio for enumeration data.

2. Statistical inference method

① Measurement data: adopt t-test, paired t-test, rank sum test, paired rank sum test, etc..

② Enumeration data: adopt pearson chi-square test, correction chi-square or Fisher exact tests; adopt Ridit analysis or CMH test for ranked data.

③ Multicenter analysis for comprehensive effect: adopt CMH test for enumeration data, and variance analysis for measurement data.

④ PP analysis and ITT analysis: adopt the two tests for overall indicators and main indicators.

3. Result expression

① The report mainly adopt statistical diagram and corresponding statistical indicators.

② All the hypothesis testing adopt two-sided test, take P≤0.05 for statistically significance.

(Ⅳ) Statistical software

Adopt SPSS15.0 for windows software for analysis.

**Ⅻ. Ethics Principle**

(Ⅰ) Ethics review system

Establish multicenter clinical research ethical review system to ensure the clinical research in accordance with Helsinki Declaration and Chinese laws and regulations relevant to the clinical research. The research are investigated by ethics committee before start of the research, and each center ethics committee for reference. Each center ethics committee fulfill the formalities, means decide whether to agree to the record or not after checked the feasibility of the research, including qualification and experience of the researchers, equipment and lab environment. Each center ethics committee must convene a meeting for investigation when serious AE happened, and notify the result to the others.

(Ⅱ) Informed consent

Informed consent are signed before the clinical research. The researchers must make the subjects clearly understand the research objectives, observed indicators, methods, examination and potential deficiency, etc.. The research would start after all the subjects sign the information consent form. The information consent form showed in accessory 2.

(Ⅲ) Benefit and risk

Western medicine treat gout in break period by xanthine oxidase inhibitor(such as allopurinol) or promote uric acid excretion medicine(such as probenecid, sulphinpyrazone, benzbromarone, and etc.) at present. There is certain curative effect, but also a lot of side-effect, such as gastrointestinal reaction, exanthema, anaphylaxis, myelosuppression, liver and kidney damage, etc.. Traditional Chinese medicine can treat gout in certain effect, and avoid or relieve the side effects caused by western medicine. So traditional Chinese medicine maybe a better treatment for gout.

(Ⅳ) Suspend research in advance

The research would suspend when the treatment have potential risk for the body prompted by the frequency and severity of AE. Before suspend, the researchers must inform the sponsor, ethics committee and State Drug Administration.

(Ⅴ) Treatment after the research

The patients completed the research can choose the same treatment or others according to their intention and condition. The researchers must adopt effective medical method in hand for the patients who appeared the serious AE.

1. **Thirteen Conclusions and file maintenance**

Original document and audiovisual materials are preserved by clinical research center special person in storage cabinets.

The researchers uploading all electronic data to Nanjing University of Chinese Medicine affiliated hospital group by data management system. The later group submit the data base for the researchers for statistical analysis.

Zhejiang Chinese Medical University and the affiliated hospital, the medical school of Zhejiang University and the affiliated hospital take charge in conclusion.

Each research centre own the data ownership, the affiliated hospital of Nanjing University of Chinese Medicine can't provide the data to a third party (only when have the written authorization from the researchers), and use the study data to publish clinical research report.

1. Fourteen Clinical research flow chart

|  | | | Screening period | Supervision period | |
| --- | --- | --- | --- | --- | --- |
|  |  |  | 0 week | 6 weeks | 12 weeks |
| Supervision serial number | | | 1 | 2 | 3 |
| Information consent form signed | | | ● |  |  |
| General conditions | | | ● |  |  |
| Primary gout family history | | | ● |  |  |
| Drug allergy history | | | ● |  |  |
| Previous history | | | ● |  |  |
| Drug used before the research* | | | ● |  |  |
| Vital sign | | | ● | ● | ● |
| Systematic physical examination | | | ● | ● | ● |
| Laboratory Examinations | Blood routine | | ● | ● | ● |
|  | Urine routine | | ● | ● | ● |
|  | Dung routine + OB | | ● |  | ● |
|  | ESR | | ● | ● | ● |
|  | Blood biochemical | UA | ● | ● | ● |
|  |  | GPT（ALT、AST） | ● | ● | ● |
|  |  | BUNCr （BUN，Scr） | ● | ● | ● |
|  |  | Blood fat （TC、TG、HDL-C） | ● |  | ● |
|  |  | GLU | ● |  | ● |
|  | ECG** | | ←―――――――――――――――――――→ | | |
|  | Urinary tract B to exceed ** | | ←―――――――――――――――――――→ | | |
|  | Others examinations** | | ←―――――――――――――――――――→ | | |
| Western medicine diagnosis | | | ● |  |  |
| Diagnosis based on traditional Chinese medicine | | | ● |  |  |
| Inclusion / exclusion criteria | | | ● |  |  |
| Results after screening | | | ● |  |  |
| Times of Joint swelling and pain recurrence | | |  | ● | ● |
| Suspend criteria | | |  | ● |  |
| Prescription and release drug | | | ← Prescribe and release drug every1-2weeks→ | |  |
| Drug combination ^△△^ | | | ←―――――――――――――――――――――→ | | |
| AE^△△^ | | | ←―――――――――――――――――――――→ | | |
| Situation at the end of research | | | Fillout at the end of research | | |

Notice: * means the drug was suspended before the research, fillout the drug in *drug combination* when nosuspended. ** means the examine when necessary, and reasoned if not necessary. ^△^ record by subject or researcher.

^△△^observed in all the process, and record when it happened.

1. **The study task allocation(table, including person in charge and the researchers) and expected research progress**

(Ⅰ) The study task allocation

| Research centre | Researcher | Task |
| --- | --- | --- |
| Zhejiang Chinese Medical University | WenChengping | Subject design、management and Organization and implementation |
|  | FanYongsheng | Clinical design and cases observation |
|  | XieZhijun | Quality monitoring |
|  | HeMiaoquan | Quality monitoring |
| First affiliated hospital of Zhejiang Chinese Medical University | LiXueming  SongXinwei | 30 cases observation |
| 2-nd affiliated hospital of Zhejiang Chinese Medical University | GaoXiangfu  WangXinchang | 30 cases observation |
| 3-rd affiliated hospital of Zhejiang Chinese Medical University | WenChengping | 66 cases observation |
| 2-nd affiliated hospital of Zhejiang University | WuHuaxiang | 30 cases observation |
| Zhejiang hospital | JinXiaoqing | 30 cases observation |
| China Academy of traditional Chinese medicine Guanganmen hospital | JiangQuqn | 30 cases observation |
| Jiangsu Province Traditional Chinese Medicine Hospital | XiongNingning | Formulate electronic cases and as the third party |

**S1 Fig.** Flow diagram of participants through the enrollment and

treatment


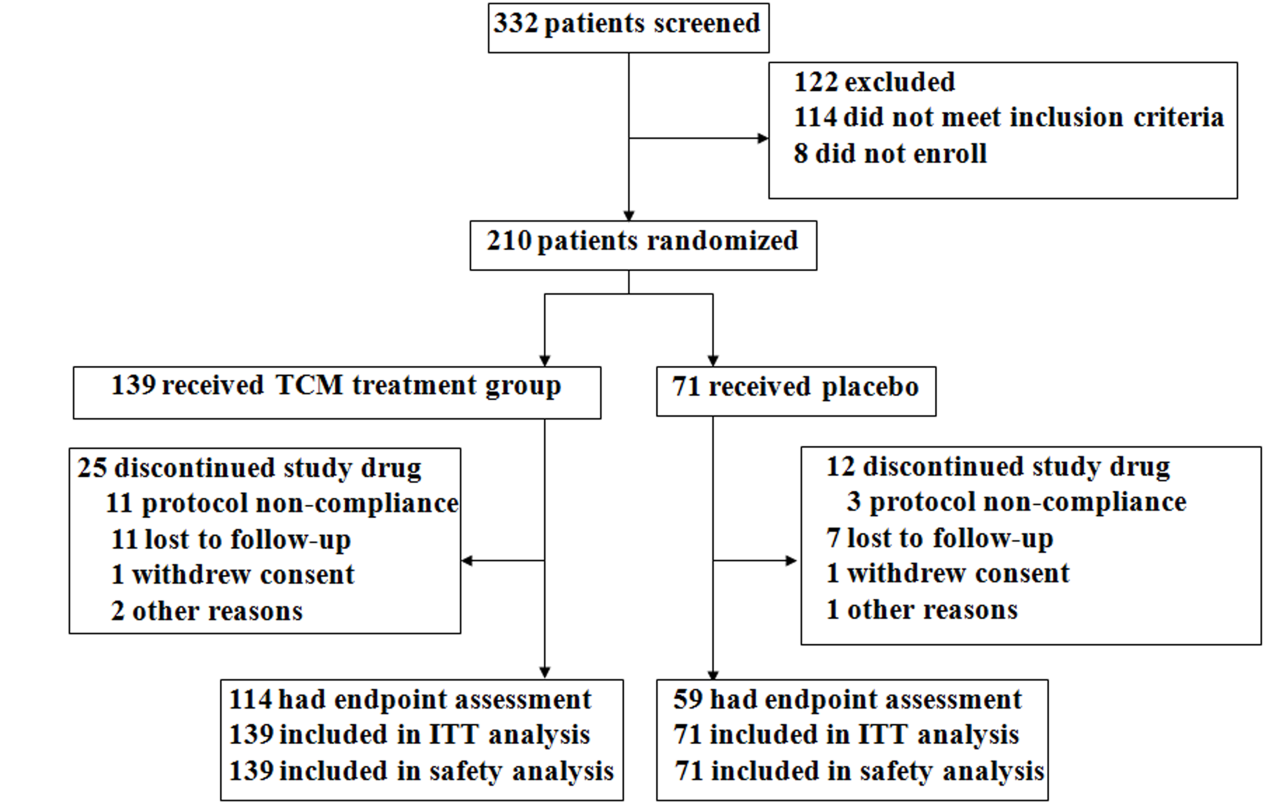


**Fig 1.** **Flow diagram** **of participants through the enrollment and treatment** Of the 332 male patients with gout screened for eligibility, 114 did not meet the inclusion or exclusion criteria and 8 did not enroll. Of the 210 patients assessed for eligibility at baseline, 139 were assigned to the treatment group and received CoTOL treatment, and 71 were assigned to the control group and received placebo. During 12-week follow-up, a total of 25 patients in the treatment group and 12 in the control group had interrupted treatment. Thus, 114 patients who underwent the complete course of treatment and had endpoint assessment were included in PPS analysis and 139 were included in ITT and safety analyses in the treatment group, while 59 were included in PPS analysis and 71 were included in ITT and safety analyses in the control group.

**S2 Fig.** Effect of CoTOL on reducing serum uric acid

**
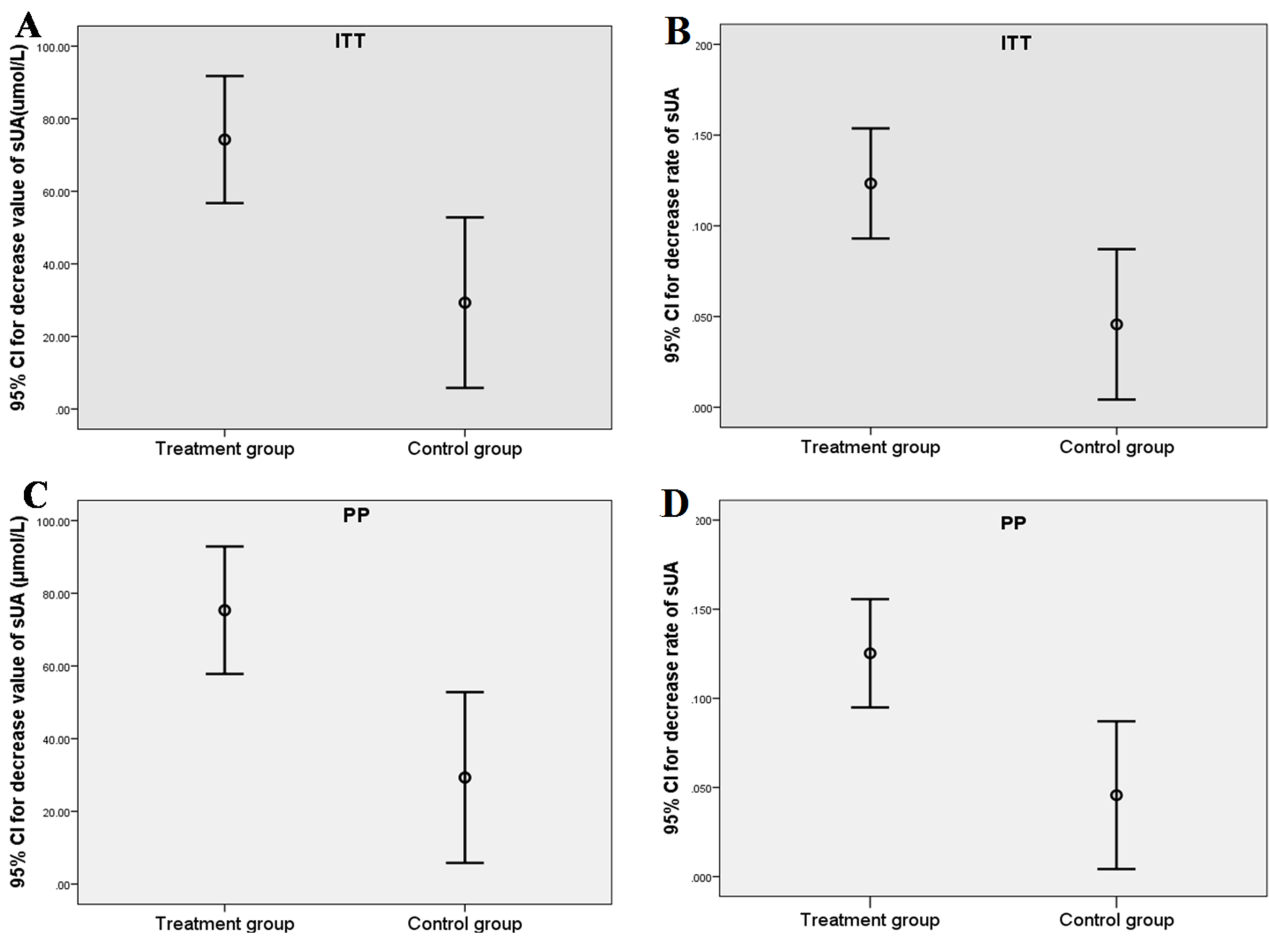
**

**Fig 2**. **Effect of** **CoTOL on reducing serum uric acid (sUA).** (A) ITT analyses for decrease value of sUA after 12-weeks treatment . The difference between the two groups was statistically significant (*z*=-2.87*,P*=0.004, Wilcoxon rank sum test). (B) ITT analyses for decrease rate of sUA after 12-weeks treatment . The difference between the two groups was statistically significant (*z*=-2.86*,P*=0.004, Wilcoxon rank sum test). (C) PP analyses for decrease value of sUA after 12-weeks treatment . The difference between the two groups was statistically significant ( *z*=-2.956*,P*=0.003, Wilcoxon rank sum test). (D) PP analyses for decrease rate of sUA after 12-weeks treatment. The difference between the two groups was statistically significant (*z*=-2.954*, P*=0.003, Wilcoxon rank sum test).

ITT

| **Ranks** | | | | |
| --- | --- | --- | --- | --- |
|  | group | N | Mean Rank | Sum of Ranks |
| 访1，3尿酸差值 | Treatment group | 113 | 93.78 | 10597.50 |
|  | Control group | 58 | 70.84 | 4108.50 |
|  | Total | 171 |  |  |
| 访1，3尿酸下降率 | Treatment group | 114 | 93.66 | 10677.50 |
|  | Control group | 57 | 70.68 | 4028.50 |
|  | Total | 171 |  |  |

| **Test Statistics^a^** | | |
| --- | --- | --- |
|  | 访1，3尿酸差值 | 访1，3尿酸下降率 |
| Mann-Whitney U | 2397.500 | 2375.500 |
| Wilcoxon W | 4108.500 | 4028.500 |
| Z | -2.870 | -2.862 |
| Asymp. Sig. (2-tailed) | .004 | .004 |
| a. Grouping Variable: group | | |

PP

| **Ranks** | | | | |
| --- | --- | --- | --- | --- |
|  | 组别 | N | Mean Rank | Sum of Ranks |
| 访1，3尿酸差值 | 1 | 112 | 93.53 | 10475.50 |
|  | 2 | 58 | 69.99 | 4059.50 |
|  | Total | 170 |  |  |
| 访1，3尿酸下降率 | 1 | 113 | 93.42 | 10556.50 |
|  | 2 | 57 | 69.80 | 3978.50 |
|  | Total | 170 |  |  |

| **Test Statistics^a^** | | |
| --- | --- | --- |
|  | 访1，3尿酸差值 | 访1，3尿酸下降率 |
| Mann-Whitney U | 2348.500 | 2325.500 |
| Wilcoxon W | 4059.500 | 3978.500 |
| Z | -2.956 | -2.954 |
| Asymp. Sig. (2-tailed) | .003 | .003 |
| a. Grouping Variable: 组别 | | |

**S3 Fig.** Effect of CoTOL on preventingrecurrence of gouty

arthritis attack


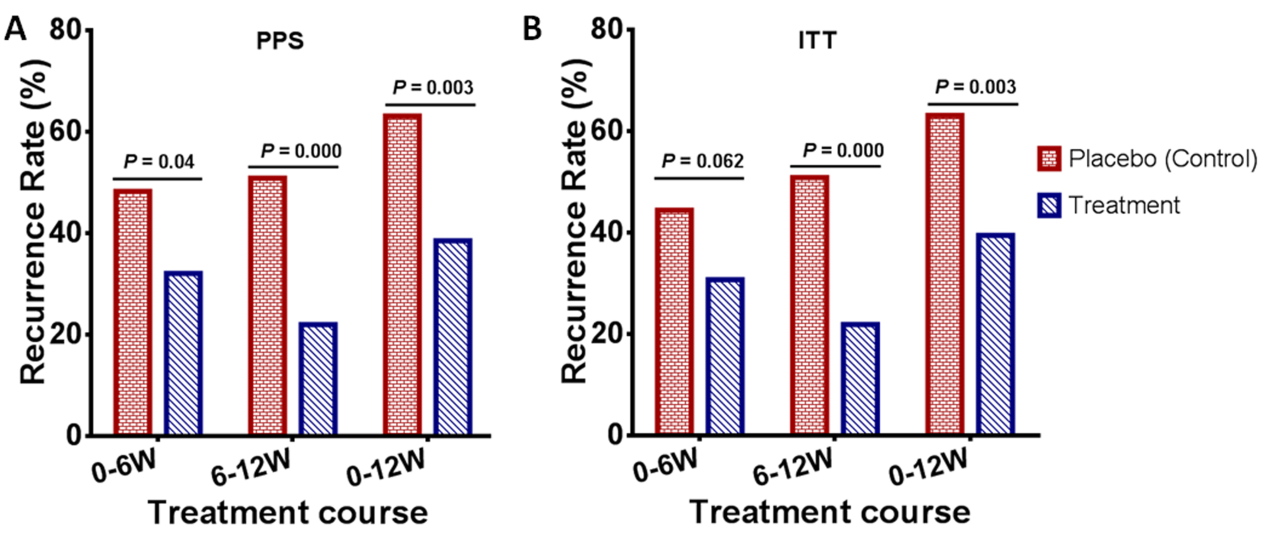


**Fig 3. Effect of CoTOL on preventing recurrence of Gouty arthritis**  (A) PPS analyses (Pearson χ^2^ test): from baseline to 6^th^ weekend, 32.14% in treatment group vs. 48.28% in control group, *P*=0.04; from 6^th^ weekend to 12^th^ weekend, 22.02% in treatment group vs. 50.88% in control group; *P*=0.000; from baseline to 12^th^weekend, 38.53% in treatment group vs. 63.16% in control group, *P*=0.003. (B) ITT analyses (Pearson χ^2^ test): from baseline to 6^th^ weekend, 30.77% in treatment group vs. 44.44% in control group, *P*=0.062; from 6^th^ weekend to 12^th^ weekend, 21.93% in treatment group vs. 50.88% in control group; *P*=0.000; from baseline to 12^th^ weekend, 39.50% in treatment group vs. 63.16% in control group, *P*=0.003.

| Table 4-1. Recurrence of Joint Swelling and Pain for PPS* | | | | | | | | |
| --- | --- | --- | --- | --- | --- | --- | --- | --- |
| Treatment | Treatment |  |  | Control |  |  |  |  |
| Course | Recurrence |  | Recurrence rate (%) | Recurrence | | Recurrence rate (%) | *χ^2^* | *P* |
| (week) | Yes | No |  | Yes | No |  |  |  |
| 0-6 | 36 | 76 | 32.14 | 28 | 30 | 48.28 | 4.237 | 0.04 |
| 6-12 | 24 | 85 | 22.02 | 29 | 28 | 50.88 | 14.342 | 0 |
| 0-12 | 42 | 67 | 38.53 | 36 | 21 | 63.16 | 9.112 | 0.003 |
| * Statistical analysis technique：Pearson *χ*2 test | | | | | | | | |
|  |  |  |  |  |  |  |  |  |
| Table 4-2. Recurrence of Joint Swelling and Pain for ITT* | | | | | | | | |
| Treatment | Treatment |  |  | Control |  |  |  |  |
| Course | Recurrence |  | Recurrence rate (%) | Recurrence | | Recurrence rate (%) | *χ^2^* | *P* |
| (week) | Yes | No |  | Yes | No |  |  |  |
| 0-6 | 40 | 90 | 30.77 | 28 | 35 | 44.44 | 3.478 | 0.062 |
| 6-12 | 25 | 89 | 21.93 | 29 | 28 | 50.88 | 14.737 | 0 |
| 0-12 | 47 | 72 | 39.50 | 36 | 21 | 63.16 | 8.659 | 0.003 |
| * Statistical analysis technique：Pearson *χ*2 test | | | | | | | | |
